# Through the looking glass: understanding non-inferiority

**DOI:** 10.1186/1745-6215-12-106

**Published:** 2011-05-03

**Authors:** Jennifer Schumi, Janet T Wittes

**Affiliations:** 1 Statistics Collaborative, Inc., Suite 600, 1625 Massachusetts Avenue NW, Washington DC 20036, USA

## Abstract

Non-inferiority trials test whether a new product is not unacceptably worse than a product already in use. This paper introduces concepts related to non-inferiority, and discusses the regulatory views of both the European Medicines Agency and the United States Food and Drug Administration.

## Introduction

'Well, in our country,' said Alice, still panting a little, 'you'd generally get to somewhere else - if you ran very fast for a long time as we've been doing.'

'A slow sort of country!' said the Queen. 'Now, here, you see, it takes all the running you can do, to keep in the same place. If you want to get somewhere else, you must run at least twice as fast as that!'

Lewis Carroll, *Through the Looking Glass*

Classical statistics is non-intuitive enough when you are trying to show that a new intervention is better than a previous one. You cannot prove what you want to prove; all you can say is that the data you observe provide sufficient evidence to reject the hypothesis that the two interventions have the same effect. Then, when you try to estimate the magnitude of the effect, all you can say is that if you repeated your experiment an infinite number of times and calculated your confidence interval (CI) as you were taught, 95% of those intervals would cover the true effect. (No wonder people flee to Bayesian inference!) But as difficult and counterintuitive as classical statistics may be, they are simple compared with the problems of inference in non-inferiority trials.

When designing a trial to show superiority of a new intervention, you specify a null hypothesis; consistent with the word 'null', your hypothesis asserts that the two interventions are the same. You then choose an alternative hypothesis stating that the difference between the means, or some other statistic, is γ. Throughout this paper, we assume that larger positive outcomes are better than smaller positive outcomes, and a positive treatment difference, γ, provides evidence of benefit. For situations in which a smaller outcome is better than a larger outcome (for example, tumor size in cancer applications) the signs in this paper would change from negative to positive. You use your chosen Type I error rate or α, your desired power, and γ to select your sample size. The goal of your experiment is to reject that null hypothesis, thus γ is in some sense a tool to help you select your sample size. At the end of the trial, the estimated effect may be bigger or smaller than γ, but as long as the lower bound of your 95% CI is above zero, you may reject your null hypothesis. The preselected γ plays no formal statistical role in the analysis of a superiority trial, although the difference in magnitude between the hypothesized γ and the estimated effect is likely to influence how to interpret the results.

**Figure 1:**
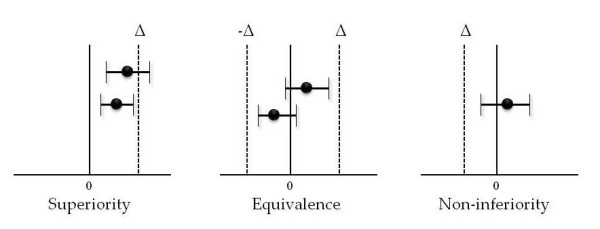
**The role of Δ in superiority, equivalence and non-inferiority trials**.

A non-inferiority experiment, by contrast, tries to show that the new intervention is not 'inferior' to the previous one, or, more precisely, that the new intervention is 'not unacceptably worse' than the intervention used as the control. Thus the null hypothesis seems backwards, in a sense, as this hypothesis is not 'null' at all. Instead, it states that the new treatment is worse than the old by more than -Δ, where -Δ is the 'non-inferiority margin'. The alternative hypothesis states that the difference in the effect between the new and old interventions is less than -Δ (Figure 1). In the inverted world of non-inferiority, the alternative hypothesis seems 'null', whereas the null hypothesis includes a specified treatment difference of -Δ. Here, -Δ is an integral part not only of the design, as with γ in superiority trials, but of the analysis as well, a role that γ does not play in superiority trials.

Reversing the null and alternative hypotheses may be the first looking-glass problem of non-inferiority, but as we peer deeper, the backwardness seems to multiply!

Trials to show superiority generally penalize the sloppy investigator (although not always; improper handling of missing data can benefit a more toxic, less efficacious treatment, potentially increasing the possibility of a false finding in a superiority trial). By contrast, non-inferiority trials tend to reward the careless. The less rigorously conducted the trial, the easier it can be to show non-inferiority. As treatments improve, showing benefit of a new therapy becomes more and more difficult, but showing non-inferiority becomes ever easier, stemming from 'lack of constancy' (deliciously termed 'biocreep' in drugs and 'technocreep' in devices). But wait! There's more! Non-inferiority trials also face the issue of 'assay sensitivity', the reality that, in some disease settings, even truly effective drugs do not always show benefit in a clinical trial. This means that a non-inferiority trial in a setting in which the standard drug would not have been shown superior to placebo would be likely to demonstrate non-inferiority of the new treatment (see [1,2] for further discussions of assay sensitivity and other issues related to active controlled trials).

For all these reasons and probably several more, many investigators faced with the challenge of designing and interpreting non-inferiority trials often despair when trying to understand them. In this commentary, we explain what a non-inferiority trial attempts to show; we amplify some of the problems discussed above; we distinguish the regulatory view of the US Food and Drug Administration (FDA) from that of the European Medicines Agency (EMA); and, perhaps, most important, we discuss why such trials are often desirable to perform.

## Superiority, equivalence, and non-inferiority

Investigators understand intuitively, even before applying statistical rigor, how to conduct a trial to establish superiority of a novel treatment. When a new therapy is compared with a placebo control, or, if one exists, an active control, the investigator defines an outcome (such as level of pain or overall survival) and declares the new treatment superior if, at the end of the trial, the estimated value of the outcome in the treated group is 'better' than the estimate in the control group. Statistically speaking, 'better' means that the data allow rejection of the null hypothesis that the two distributions are equal, in favor of the hypothesis that the new treatment is better than the control.

Sometimes, the goal is not to show that the new treatment is better, but that the new treatment is 'equivalent' to the control. Because only with an infinite sample size would it be possible to show exact equivalence, investigators instead select a margin. Again, call it Δ. At the end of the trial, a CI is computed around the difference between two test statistics (equivalence trials typically use 90% CIs) and if the CI lies strictly within [-Δ, +Δ] the two treatments are called 'equivalent.' Such trials are used to show that a generic drug is biologically the same as the drug it is trying to mimic. They are also used to show lot consistency in vaccine trials, in which the outcome is a measure of immune response.

Non-inferiority is different from equivalence. In an equivalence trial, the desired conclusion is that two products are the same or 'not unacceptably different' from each other. In a non-inferiority trial, by contrast, the aim is to show that a new product is not unacceptably worse than an older one. Why might it be reasonable to pursue a product that is possibly less efficacious than an existing therapy? A new treatment that is not much worse than, or 'non-inferior to', the standard treatment may be attractive if, when compared with the standard treatment, it is expected to cause fewer side effects, or lead to improved quality of life, or if its dosing regimen is easier to tolerate.

Assume it is possible to define what 'significantly worse' means (think of this as a window of indistinguishability, or a margin that we will call -Δ; below we discuss how to choose such a margin), and that there is an existing treatment available against which to compare the new treatment. The new treatment could be said to be not unacceptably worse than [3] (that is, non-inferior to) the existing treatment if, when the CI around the difference in the effect size between the new and existing treatments is calculated, the lower bound of that interval does not extend beyond the window of indistinguishability defined above. One focuses on the lower bound for this non-inferiority comparison; what happens at the upper end of the CI is not the primary concern. In an equivalence trial, by contrast, investigators care about both ends of the CI, and would declare the new treatment equivalent to the existing treatment only if the entire CI falls within this margin on either side of zero.

Non-inferiority trials are clearly appropriate for some diseases and some treatments. When developing a new treatment to prevent tuberculosis, investigators might be willing to sacrifice some small amount of benefit (as reflected in the margin) for a simpler dosing schedule, fewer side effects, or other advantages, but they would be delighted if the new treatment were better than current therapies (hence no restriction on the upper bound of the interval) and they could also declare superiority. This would only happen if the lower bound of the interval were above zero, not simply above -Δ.

Thus far, the problem sounds straightforward. One needs to select a non-inferiority margin, run the trial comparing the experimental treatment to an active control, calculate the CI around the difference between the treatments, and examine the lower bound of the CI. If the lower bound is above the margin -Δ, the new treatment is deemed non-inferior, and the trial is a 'success'. Further, if the new treatment is statistically significantly better than the comparator (that is, the lower bound of that same CI is also above zero), then superiority of the new treatment can also be declared. Importantly, testing first for non-inferiority and then for superiority does not require a statistical 'penalty' for multiple testing, because testing first for non-inferiority before testing for superiority (while examining a single CI) uses a testing procedure that appropriately controls the overall Type I, or α, error rate of the two tests. Statisticians refer to this type of testing as 'closed testing', and such a process ensures that the overall experiment-wise error rate is maintained at the correct level when testing more than one hypothesis. The order of the testing is important; to declare superiority, a new treatment necessarily also has to be declared non-inferior. The converse (testing first for superiority and then for non-inferiority) is not always a closed procedure. Testing in that order could lead to apparently anomalous results, even when examining a single CI. A large trial with a narrow CI around the difference between the active control and the new treatment might show that the lower limit of the interval lies within the margin, meaning that the new treatment is non-inferior to the active control, but the upper limit of the interval is below zero, so the new treatment is also inferior to the active control. Bear in mind that the opposite of 'non-inferior' is not 'inferior'; it is the looking-glass opposite, 'not non-inferior'. As an example, suppose the margin -Δ is -3, and the observed 95% CI at the end of the trial is [-2.7, 1.5]. The lower limit of the CI is above -3, so the new drug is non-inferior to the old, but the upper limit of -1.5 is less than zero, so the new drug is also inferior to the old. In this case, the single CI can be used to say that the new treatment is simultaneously 'non-inferior' and 'inferior'. Although this example may seem counterintuitive, when interpreting the results of a non-inferiority trial, it must be remembered that the purpose of the trial is to estimate the lower bound of the CI, not to establish a point estimate of the treatment effect. This test, sitting on the other side of the looking glass, requires an interpretation different from the usual.

In some trials, it is statistically appropriate to perform a superiority comparison first and, if that does not show statistical benefit, to perform a non-inferiority comparison. That would be appropriate only when the non-inferiority margin had been preselected. The reason such a switch is permissible stems from the fact that we can view the test as an interpretation of a CI. The calculated CI does not know whether its purpose is to judge superiority or non-inferiority. If it sits wholly above zero, then it has shown superiority. If it sits wholly above -Δ, then it has shown non-inferiority.

**Figure 2:**
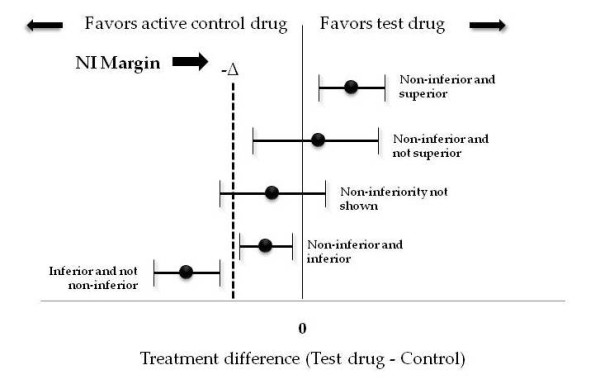
**Possible outcomes of a non-inferiority trial**.

A non-inferiority trial can have five possible types of outcomes as depicted in Figure 2. The two vertical lines indicate zero and -Δ. Each horizontal line represents a CI, with the estimated treatment effect denoted by the dot in the center. The CI at the top of the figure sits wholly above zero; a trial with this outcome would conclude that the new treatment is superior and hence, also non-inferior, to the control. The next interval, which spans zero but lies wholly above -Δ, represents a trial that has shown non-inferiority, but not superiority. The third interval, which straddles both zero and -Δ, represents a trial that has shown neither non-inferiority nor superiority. The fourth CI illustrates the case discussed above; tucked between the two vertical lines, it shows both non-inferiority (because it lies wholly above the line for -Δ) and inferiority (because it also lies wholly below zero). The final CI on the bottom of the figure shows inferiority and does not show non-inferiority.

## Complications - other than the margin

Among the challenges in non-inferiority trials compared with superiority trials are the choices of the margin, the primary population for analysis, and the comparator treatment. As in our previous section, we delay discussion of the margin and tackle the latter problems first.

Conventional wisdom suggests that in a non-inferiority trial, the primary population for analysis should be the per-protocol (PP) population, which in this case is the set of people who have taken their assigned treatment and adhered to it. (Recall that superiority trials use the total, or intention-to-treat (ITT), population for the primary analysis.) Many appeal to the PP population in a non-inferiority trial because the more poorly run a trial, the more likely an ITT analysis will show non-inferiority. Consider a trial with a hopelessly flawed randomization, where instead of creating two distinct treatment groups (one set of subjects receiving the new treatment and the other the active comparator), the randomization scheme actually created two 'blended' groups, each composed of half subjects receiving the new treatment and half receiving the active comparator. If this trial were testing for superiority, the test would, with high probability, correctly find no difference between the groups. As a non-inferiority trial, however, such a flawed trial would be very likely to incorrectly demonstrate non-inferiority. This trial as described is an extreme example of the importance of assay sensitivity, in that a trial with such a flawed allocation scheme has lost the ability to distinguish any true differences between treatment groups that may exist, and is an argument for why conventional wisdom favors showing benefit in the PP population.

Others [4] (including the authors) disagree with that opinion. Appeal to the dangers of sloppiness is not a reason for using the PP population but rather a reason for ensuring that a trial is well designed and carefully monitored, with the primary analysis performed on an ITT population. From a regulatory perspective, however, both populations are of interest. The US and European regulators are interested in success on both the ITT and the PP populations. The EMA publication *Points to Consider on Switching between Superiority and Non-inferiority *[5] specifically states that a non-inferiority trial must show non-inferiority in both the ITT and the PP populations. The US regulators [6] cite 'significant concerns with the possibility of informative censoring' in an 'as-treated' or PP, analysis, and advise investigators to plan both types of analyses in their non-inferiority trials. They go on to state that discrepancies between the two types of analyses will require 'close examination', words that no investigator wants to hear from regulators.

An investigator may also have several choices for the comparator arm in a non-inferiority trial, but it must be a 'fair fight'. One example of an unfair control would be a comparator with a dose that is lower than optimal. Another stems from biocreep. Suppose an earlier trial found drug A to be clearly better than placebo, then several years later, drug B is found non-inferior to drug A in a trial with a large non-inferiority margin. Drug C is then compared to drug B, again with a large non-inferiority margin, and shown to be non-inferior to B. This is an example of biocreep; at each step, the new drug has been shown to be not unacceptably worse than the previous. Hence, a comparison of a new drug with drug C may not be fair, because drug C may in fact be less effective than drug A and, if the margins were too big, even less effective than placebo. We mention this situation again below when talking about 'constancy.'

Sufficient data need to be available to allow calculation of the non-inferiority margin for the same disease and endpoint. The FDA Guidance [6] does allow, however, that the active control need not be approved for the indication of interest in the non-inferiority trial if these data exist.

## Choosing the margin, conceptually

Having agreed to analyze both the ITT population and some version of a PP population, and having selected the appropriate active control, an investigator next must select the non-inferiority margin and method of analysis. One approach would be to ask clinicians or patients to consider what degree of efficacy they would be willing to sacrifice in exchange for the potential benefits offered by the new treatment. A panel of clinical experts with knowledge of existing treatment options and underlying disease may be able to consider trade-offs on the level of the patient population, and could propose a plausible non-inferiority margin. Patient groups could perhaps provide more insight into trade-offs that potential patients might be willing to make for a product with benefits such as an improved dosing schedule or fewer side effects. Such an argument, seeking guidance from the oracle of clinical judgment or patient experience, may be appealing from the perspective of some physicians, but such a Delphic method may have limited success in a scientific or regulatory setting, which could require justification of expected treatment effects and variability.

Two more formal approaches to the margin and analysis are the putative placebo (also known as synthesis method) and the 95-95 approach in Rothmann [7,8].

The 95-95 method starts by calculating M_1_, the entire effect of the active control relative to placebo. This calculation typically uses meta-analytic methods with data from previous studies, as we describe below, to obtain a 95% CI around the estimated difference between the active control and placebo. A conservative estimate of this difference, the lower limit of that CI, is then used as M_1_. Next, a smaller margin, M_2_, is specified in order to preserve some predetermined fraction of the estimated active control effect, for example, 50 or 75%. We can interpret M_2 _as the largest loss of effect (inferiority) that would be clinically acceptable when comparing the test drug to the active control. These definitions of M_1 _and M_2 _come from the notation used in the FDA Guidance document, which we discuss in the next section. Having established the margin M_2_, a non-inferiority trial using the fixed-margin approach is successful if the lower limit of the 95% CI around the difference between the new treatment and the active control lies above that margin.

The synthesis method, by contrast, does not require specification of a specific margin or active control effect [6,9]. This approach specifies a threshold for the desired fraction of the effect of the active control that is retained by the new treatment. Thus, the test of the non-inferiority hypothesis in this type of analysis is based on a combination of the estimate and standard error (SE) for the comparison of active control with placebo, which is not observed in the current study, and the estimate and SE for the comparison of the new treatment with the active control in the current study. This method assumes that the effect of the active control remains reasonably constant over time, or that if the effect does diminish over time (as a result, for example, of improved concomitant therapies), such a modified effect can be estimated. See Rothmann *et al*. [7] for insights into modeling either the active control effect or its variability, and the papers by Snappin and Jiang [10,11] for a unified approach to both the fixed-margin and synthesis approach, which addresses the assumptions of assay sensitivity and constancy, and their implications on the Type I error rate. We amplify these concepts below in the section on technical issues.

## Regulatory perspectives

In March 2010, the Centers for Drug Evaluation and Research (CDER) and Biologics Evaluation and Research (CBER) of the US FDA issued a draft *Guidance for Industry *on non-inferiority trials [6]. FDA Guidance documents represent the Agency's current thinking on a wide variety of topics in the drug development process, including clinical issues, statistics, manufacturing, safety, and labelling. This Guidance opens with an introductory overview of non-inferiority trials. It then provides a careful discussion of statistical issues, including methods for determining an appropriate non-inferiority margin, and closes by addressing questions through illustrative examples from recent submissions. Much of the philosophy underlying this Guidance deals with the concern of the FDA that in a trial without a placebo group (or more generally, an untreated control), failure to find a difference between the new treatment and the active control may actually mean that neither would have been better than placebo. Thus, one way to look at the Guidance is to consider it an attempt to ensure that a study that concludes 'non-inferiority' has identified a treatment that is superior to placebo.

The Guidance provides useful notation, which we have adopted for our discussion in this paper. As described above, we use M_1 _to denote the entire effect the active control has relative to placebo, and M_2 _to denote the largest loss of effect (inferiority) that would be clinically acceptable when comparing the test drug with the active control.

The effect M_1 _is calculated from historical information; it is not measured directly in a non-inferiority trial (unless the trial includes a third arm, either a placebo or no treatment). Nevertheless, the assumed advantage of the active control over placebo must also be present in the current study, even if the advantage is not directly observed. We will discuss this assumption, known as 'assay sensitivity', in greater detail below.

The Guidance notes that in certain settings, it may be reasonable to demonstrate only non-inferiority to the M_1 _margin. Such a result shows that the test drug has a non-zero effect, but that effect may not be clinically meaningful. The smaller margin, M_2_, tightens the connection between the test drug and the active control, allowing a claim of non-inferiority only if the test drug has not lost 'too much' of the effect of the active control. The Guidance states:

'...a successful non-inferiority study shows rigorously that the test drug has an effect greater than zero if it excludes a NI margin of M1, so long as M1 is well chosen and represents an effect that the control drug actually would have had (versus a placebo, had there been a placebo group). It can also show that the test drug had an effect greater than some fraction of the control drug effect, depending on the M2 that is used.' ([6], page 12, section III.B).

Although non-inferiority trials are often conceptually desirable, operational difficulties may plague the process of choosing the margin, either because of insufficient data to support a selected margin or a calculated margin that leads to an impractical sample size. The Guidance briefly discusses alternative designs that may be preferable in these situations, including add-on studies, carefully selected patient populations, or randomized withdrawal studies.

The main focus of the Guidance comes in section IV: 'Choosing the non-inferiority margin and analyzing the results of an NI trial'. Conceptually, the Guidance breaks down the process into two steps: determining a reasonable way of assessing the effect of the active control in the current study, and then showing that the benefit of the active control over the test drug in the current study is no greater than the (assumed) entire effect of the active control over placebo.

Once the margin has been selected, either from expert opinion or from formal analytics of historical data, the designers of the trial must determine how to analyze the results of a non-inferiority study. The Guidance lays out two possible approaches, which we have briefly introduced above: the fixed-margin method (also known as the double CI method or the 95-95 method [7,8]) and the synthesis method. In the fixed-margin method, a non-inferiority trial 'succeeds' if the lower limit of the 95% CI around the difference between the test drug and the active control sits above the margin, either M_1 _or M_2_.

By contrast, the synthesis method does not define a specific margin or effect of the active control based on past trials. The Guidance says that 'the synthesis method is designed to directly address the question of whether the test product would have been superior to a placebo *had a placebo been in the NI study *[emphasis ours], and also to address the related question of what fraction of the active comparator's effect is maintained by the test product' ([6], page 30). This approach combines the effect of the test product that is observed in the non-inferiority trial with an estimated control effect, from either a single trial or a meta-analysis, to obtain a single CI that is used to test the non-inferiority hypothesis comparing the test product with the active comparator. Considered another way, however, the synthesis method could be applied (under the setting of 0% retention of the active control effect) to test whether or not the test product is better than placebo, assuming that an unbiased estimate can be obtained of the active control effect relative to placebo. The most important assumption here is that the effect of the active control has remained relatively constant (or can be modeled as discussed above) from the past into the current non-inferiority trial. This method is slightly more efficient in the statistical sense (in terms of requiring a smaller sample size to have the same statistical power), but is sensitive to assumptions, and does not readily incorporate clinical judgment into the definition of M_2_.

The Guidance concludes with responses to a series of questions commonly asked about non-inferiority trials and some examples. The questions concentrate on the choice of margin and the distinction between M_1 _and M_2_, the suitability of the active control, and options when a non-inferiority trial is not feasible. The examples illustrate the difference between the fixed-margin and synthesis approaches to analysis, how to estimate the active control effect in the absence of randomized placebo-controlled trials, a situation in which the historical active control effect is so small that a non-inferiority trial would be impractical, and a case in which the non-inferiority criteria for success can be relaxed when two studies provide consistent results.

By contrast, the EMA Guidance document on choosing a non-inferiority margin [9] does not specify a method for selecting the margin. Instead, the EMA directs trial sponsors to use a combination of statistical and clinical judgment. The method of selecting a margin could come from a Delphi-type approach asking experts how much benefit over placebo they are willing to forego by using the new product instead of the product already shown to be effective. Alternatively, investigators may choose a margin using a more formal approach. The document warns, however, that the margin selected must be sufficiently small to ensure that the experimental therapy is better than placebo. In the words of the EMA, 'a minimal requirement for the decision making process involved in interpreting data from a non-inferiority trial is that we must be confident that the test product would have been shown to be efficacious if a placebo-controlled trial had been performed'.

## Choosing the margin, technically

Whether a Delphic method, the synthesis method, or the 95-95 approach is used, the first step in defining the non-inferiority margin is to gather all relevant information about the effect of the active control. For the Delphic method, 'all relevant information' may reside in the minds, the experience, and the judgment of expert clinicians. For the synthesis and 95-95 methods, 'all relevant information' comprises the set of data addressing the magnitude of the effect of the control treatment compared with placebo. Both of these latter methods may use the same approach to identify the effect of the control relative to placebo.

### The first 95% (or how does the control compare with placebo)

As described above, the purpose of the first 95% in the 95-95 method is to calculate the effect size for the control group that gives a reasonable assurance of being no less than the true effect size. The philosophy is that calculating the 95% CI for the estimated effect size, and then choosing the lower end of that interval gives 95% confidence that the true effect size for the control intervention relative to placebo is at least as great as the calculated effect size. Having accepted this principle as the path to calculation, the next decision is what data to use to compute that CI. The FDA Guidance suggests applying meta-analytic techniques to calculate the estimated effect size and therefore the lower limit of the CI. For convenience in exposition, we discuss here binary outcomes; much of the discussion is relevant to other types of outcomes as well.

Meta-analysis is a set of methods used to combine data from a group of studies to obtain an estimate of a treatment effect. Thus, the first step in performing a meta-analysis is to collect the group of studies to use. When designing a non-inferiority trial, under ideal conditions the investigator would select a set of studies that includes only randomized trials comparing the control intervention with placebo. The patient population should be similar to the population being studied in the non-inferiority trial being planned; the outcomes studied in the trials should be the same as that planned; the control regimen (intervention and dose) should be the same as the regimen to be used in the new trial; and the current standard of care should be the same as the standard of care in the previous trials (the 'constancy' assumption). Furthermore, the total population studied in the set of trials under consideration should be sufficiently large to produce a precisely estimated effect size.

In practice, limitations of available data often force investigators to compromise on some of these criteria. The populations studied in the previous trials may differ in important ways from the population planned for the new trial. The former trials may not all have uniformly compared the control intervention to placebo; some of the trials may have used placebo whereas others may have used standard of care, and some might have used another active control. The outcome measures in the previous trials may differ from the outcome in the trial being designed. The intervention in the previous trials might have used different doses from that being contemplated in the new trial, or the relevant trials might have used a drug from the same class as the planned control, but not the same drug. And perhaps the most vexing problem of all, because it is essentially unmeasurable, is the possibility that the standard of care has changed in the years between the time of the previous trials and the trial being planned. If so, a drug shown to be effective in the past would perhaps not be shown to be effective were the same trial performed today. Similarly, if the trials under consideration for the meta-analysis were performed in countries with very different standards of care from the country in which the non-inferiority trial is to be performed, then the effect size of the control may be different from what it would have been in the country for which approval is being sought.

Assuming that the set of trials being considered do not egregiously violate the ideal standards mentioned above, the investigators are ready to produce an overall estimate of the effect size.

A meta-analysis comparing treatment A with treatment B starts with *T *randomized trials. If the primary outcome of the trial is binary, for *k *= 1, 2, ... *T*, trial *k *has sample sizes *n_kA_*and *n_kB_*with *S_kA_*and *S_kB_*successes, respectively. The outcome of the Mantel-Haenszel (MH) method is the pooled odds ratio across the *T *trials. Each study can be represented by a 2 × 2 table with the structure depicted in Table 1.

**Table 1:** Illustration of a 2 × 2 table for the *k*th trial.

	Treatment group		Total
	
	A	B	
Success	*S_kA_*	*S_kB_*	*S_k_*
Failure	*F_kA_*	*F_kB_*	*F_k_*
	*n_kA_*	*n_kB_*	*n_k_*

The odds ratio in each study (and table) is:

and the estimated MH odds ratio is [12]:

A method of Peto described by Yusuf [13] is also often used in these settings. The method differs slightly from the MH approach; however, for large sample sizes, the two methods yield almost identical results.

In both the MH and the Peto methods, the logarithm of the odds ratio under the null hypothesis is approximately normally distributed, with mean zero and variance estimated from the observations. Both methods weight studies according to their sample size, not the size of the treatment effect within the study. In other words, large studies have a large influence on the pooled effect size, while small studies have a small influence on the estimated effect.

Furthermore, if the true effect size is in fact identical in all of the studies, then the MH test is the optimal procedure, in the sense that it has the highest statistical power of all possible unbiased tests. This property is often subverted by saying that these tests require that the studies have the same true effect size, or that they are 'fixed effects models.' In fact, neither the MH nor the Peto method requires identical effect sizes. The logical interpretation of a meta-analysis using either of these methods is not that the true effect of the treatment is the same in all situations, but rather that the overall estimate obtained from a meta-analysis is the best estimate of the treatment effect, averaged over all studies included.

The FDA Guidance suggests a preference for so-called 'random-effects models' in meta-analyses that will be used to establish the margin in non-inferiority trials. These models, in contrast to the MH and Peto approaches, make very specific assumptions about the distribution of the effect size across all potential studies. The standard method, introduced by DerSimonian and Laird [14], assumes that the effect size (which in the case of binomial variables is the log odds ratio) comes from a normal distribution with mean μ and variance σ^2^. This assumption implies that the estimated pooled effect is a weighted average of the effect obtained in each study; in contrast to the MH and Peto methods, the weights are a function both of the sample sizes of the various studies and the closeness of each within-study estimate to the estimates from the other studies. As Petitti [15] points out, when results from studies are heterogeneous, random-effects models tend to overemphasize the importance of small studies. Such weighting may be inappropriate; small studies are often conducted at a single center, and are more likely to be subject to bias and less likely to have had rigorous checking of data quality or the use of rigorous methods in study conduct. See Teo *et al*. [16] for a discussion of a random-effects meta-analysis on the use of magnesium, which led to erroneous results. In that case, one small study, with results quite different from other, larger, trials, dominated the estimated effect size because the assumptions of the random-effects model put undue weight on the small trial.

**Figure 3:**
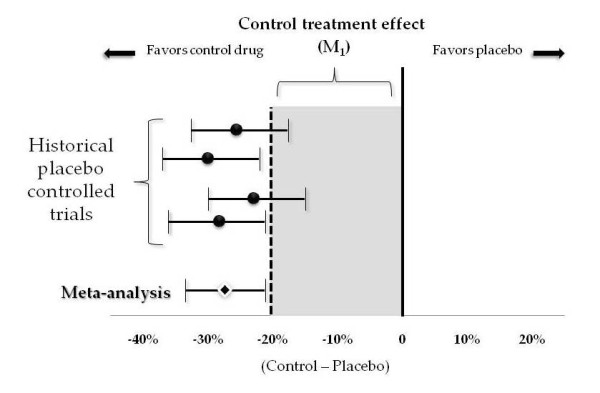
**A forest plot and M_1_**.

The typical presentation of a meta-analysis shows a forest plot depicting the results of each trial, and then a summary statistic showing the estimated effect. Having completed this meta-analysis, the investigator calculates the 95% CI and finds what FDA calls M_1_, the effect size of the control therapy that will be assumed (Figure 3).

If the outcome is a time-to-event variable or a continuous variable, the meta-analysis is typically performed on the estimated hazard ratios or means, respectively.

### Choice of M_2_: how much are we willing to lose?

As the EMA Guidance document stresses, both statistical and clinical judgment should play into the choice of margin. M_1 _is calculated, as described above, as the lower end of the 95% CI around the best estimate of the effect size of the control group relative to placebo. This number becomes the starting point for the determination of the margin. The investigator must now ask how much of that benefit is acceptable to lose if the new therapy is adopted. The past experience of the investigators may allow them to define the magnitude of a loss of efficacy that they would be clinically willing to accept. By thinking through a population of, for example, 100 cases, a clinician may be able to quantify such judgments by considering what might be an acceptable loss of efficacy compared with a standard treatment.

Sometimes, investigators do not carry out such a formal analysis; instead they figure out how much money they can spend. From there, they determine the largest trial that they can run, and justify the margin after the fact. This (not exactly a secret) is what investigators often do for superiority trials; the difference is that the purpose of a superiority trial is to show benefit, and if the power is too low for a given sample size, the trial is unlikely to show superiority. In the looking-glass non-inferiority world, however, the analogous action is to make the margin too big, increasing the chance of successfully demonstrating non-inferiority of the new treatment.

**Figure 4:**
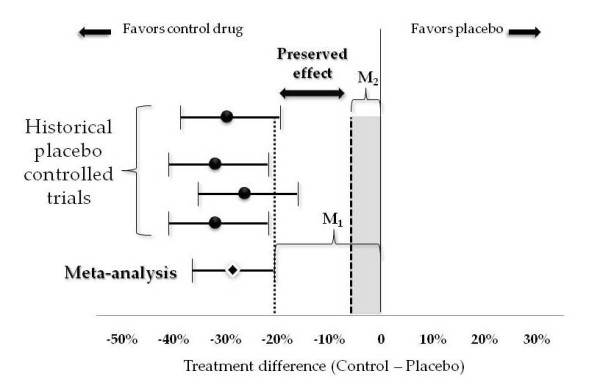
**M_2 _as a fraction of M_1_**.

M_2 _is often selected to preserve half of the effect of M_1_; however, when a drug is highly effective, losing half its effect, even though it may still be better than placebo, may not be clinically acceptable (Figure 4). Consider, for example, a childhood vaccine that prevents 96% of potential cases of disease. A new oral vaccine that only prevents 48% of disease would still be much more effective than placebo, but would hardly be attractive, even if less painful to the child than a shot. Thus, highly effective products for serious diseases should generally be evaluated in trials in which the margin preserves a large proportion of M_1. _In other settings, if the benefits of the new product, in terms of adverse events, ease of administration, and cost are very great, investigators might be willing to forego an even higher percentage of M_1_.

### The second 95% (or, is the new product non-inferior to the old?)

Having selected M_1 _(from the first 95%) and M_2 _(from judgment), the trial begins. At the end of the trial a 95% CI is calculated from the observed data. If that interval sits completely above the prespecified -Δ, the trial has shown non-inferiority. In fact, we can refer back to Figure 2 and see how the confidence limit compares with the limits shown in the figure.

## Sample size

The sample size for a non-inferiority trial is calculated to satisfy the following equation:

In words, this means that the sample size must be large enough so that the probability is sufficiently high that the lower bound of the 95% CI for the estimated difference between the treated group and the control group  is greater than the margin, -Δ, when the true difference between the groups, θ_T_-θ_C_, is γ.

Sample size for a non-inferiority trial is usually calculated under the assumption that the experimental agent and control treatment have equal effects, that is, when γ is assumed to be zero. Under the assumption that the new treatment is a little better, as is often the case for a new product, the required sample size decreases considerably.

Consider, for example, a comparison of two proportions as illustrated in Table 2. For a fixed margin, set to be 10% of the true proportion in the active control, the table presents the approximate sample size required assuming equal treatment effects, a small (5%), and a larger (10%) benefit for the experimental agent.

**Table 2:** Approximate sample sizes required for non-inferiority comparison of proportions

True proportion in active control	Non-inferiority bound using 10% margin	Approximate sample size per group assuming 1:1 randomization to new treatment and control required under:		
		
		Equal effects	5% benefit	10% benefit
0.1	0.09	19,200	8,725	5,050

0.2	0.18	8,500	3,900	2,250

0.3	0.27	4,970	2,260	1,300

0.4	0.36	3,200	1,450	825

**0.5**	**0.45**	**2,100**	**1,000**	**550**

0.6	0.54	1,440	640	360

0.7	0.63	930	405	225

Sample sizes calculated using Pass 2008 methods for non-inferiority tests of two independent proportions, using the Z statistic with continuity correction and pooled variance, with a target power of 90% and α level of 0.025.

As an example of how to read the table, consider the row in bold font, in which the true proportion in the active control is 50%. The smallest proportion that would be considered not non-inferior is 45% (a loss of 10% from the active control effect). Assuming that the proportions in the new treatment and the active control are equal, the total sample size required would be approximately 2,100 per group. If, however, the new treatment actually provided a 5% benefit over the active control, corresponding to a true proportion of 52.5%, the required sample size would be approximately 1,000 per group to show non-inferiority. That is, with a sample size of 1,000 per group, if the true proportion in the active control is 50% and the true proportion in the new treatment is 52.5%, then the probability is 90% that the lower bound of the CI is above -5%. A 10% benefit, corresponding to a proportion of 55% in the new treatment, would require a sample size of just over 500 per group to show non-inferiority.

Assuming a small benefit of the experimental agent compared with the active control cuts the sample size required roughly in half; if the larger benefit is more realistic, the sample size is roughly a quarter of that required for the assumption of equal treatment effect.

These are still, however, relatively modest improvements over the effect of active control, and although the sample size reductions when assuming these benefits are non-trivial, they are not so large as to suggest switching to a superiority trial to prove these benefits. The sample size required for a superiority trial to demonstrate the small benefit would be nearly 10 times larger than required for the non-inferiority trial, and around four times as large for the larger effect.

## Concerns about non-inferiority trials

Non-inferiority trials have a host of complications. A serious concern, as briefly described above, is assay sensitivity, the ability of a trial to distinguish an effective therapy from one that is not effective, and the issues differ for non-inferiority trials and superiority trials. A superiority trial that lacks assay sensitivity will probably show that the new therapy does not have a statistically significant benefit over control, as the trial will be unable to declare efficacy. By contrast, a non-inferiority trial without assay sensitivity may generate a positive result (that is, it may show evidence of non-inferiority) if it shows no difference between the treatment groups, as this would lead to a conclusion of non-inferiority. Unlike superiority trials, non-inferiority trials have no internal check on assay sensitivity. (The check in a superiority trial is showing that the tested intervention is superior to control.) The EMA, in an effort to mitigate this problem, has suggested that non-inferiority trials, wherever possible, include a placebo arm to allow a direct comparison of both the active control and experimental agent with placebo. (Note that the study may be the new drug, the old drug, and the placebo, all on a background of standard of care.) In many cases, such a trial is not ethically acceptable. That is, randomizing participants to placebo may not be appropriate when an existing therapy with a proven survival benefit exists (for example, in cancer), whereas in other cases (for example, pain relief) a three-arm trial could work well.

Another concern specific to non-inferiority trials pertains to the evolving standard of care, as discussed above. Consider the situation with an existing drug (drug A) that is approved for the treatment of an infectious disease on the basis of a placebo-controlled trial. Now suppose that a company applies to regulatory agencies for approval of a new treatment (drug B) using a non-inferiority design with drug A as the active control. Suppose that the trial is 'successful,' that is, drug B is shown to be non-inferior to drug A with respect to the cure rate. Presumably, if drug B has some advantages, such as fewer side effects or an improved dosing schedule, it will then become the standard of care. Then suppose the next company applies for approval of another drug (drug C) using a non-inferiority comparison against drug B. If drug A were actually not superior to placebo in the first trial, it could be fairly easy to show that each new drug is non-inferior to the active control, even when none is any better than placebo. In most cases, the issue with standard of care is not as dire as this illustration might suggest, as the point estimates could show a positive effect even if the margin allowed some loss of efficacy, but the concern is valid. As mentioned earlier, this change in effect is termed 'biocreep' in the case of drugs, and 'technocreep' in the case of devices.

Further, in the case of infectious diseases, the organisms themselves might evolve, leaving us with the possibility of true biological 'biocreep'. That is, over time, organisms develop resistance to earlier drugs in the pharmacopoeia, meaning that each new drug is being compared with an active control that might be becoming less and less effective against a strengthening infectious agent. Here, biocreep represents actual biological change in the organism. What is usually called 'biocreep' is more precisely 'virtual biocreep,' where each successive product may be a little bit less effective than the previous product [17,18].

## But what if a non-inferiority trial cannot be performed?

As alluded to above, a variety of reasons may render a non-inferiority trial unfeasible. A rigorously calculated margin could yield a sample size that cannot be supported financially or by the potential study population. The EMA Guidance specifically warns investigators not to increase their non-inferiority margin when the scientifically derived margin produces an impractically large sample size. Sometimes the necessary data may not exist (or may not be available to a new investigator) to calculate a margin as carefully as desired; or the treatment landscape may have changed so much since the historical data were collected that it is unclear what active control to use and whether or not that control really does show a benefit over placebo; or the trial may be in a therapeutic area in which well-known effective treatments do not always beat placebo (for example, depression), making it difficult to argue for the assay sensitivity required to plan a non-inferiority trial. Although challenging, such circumstances offer opportunity to the creative trialist (and statistician).

## Conclusions

A non-inferiority trial is reasonable when a new treatment has some property sufficiently favorable that physicians, and their patients, would be willing to sacrifice some degree of benefit relative to an already approved therapy. The advantage could be reduced cost, improved ease of use or dosing schedule (monthly versus weekly injections), simpler storage (not requiring refrigeration), or an improved safety profile. The benefit given up in exchange for these advantages, however, should not be so large that patients and physicians are not willing to use the new product. As discussed in the vaccine example above, an oral formulation that loses half the protection provided by an injection would not be a viable product. The choice of the non-inferiority margin and how much of the existing treatment effect to preserve incorporates in some sense these other aspects of treatment 'viability'.

From the perspective of regulators in the USA, however, success in a non-inferiority trial cannot formally incorporate these multi-faceted aspects; it simply is not the way their regulations are written at this point. The M_2 _does provide some room for flexibility by varying the proportion of the active control effect that is preserved. For serious diseases with known and highly effective treatments, any new product would need to preserve a large amount of the known treatment effect to be considered successful. In other settings (mild headache, for example), a more modest preservation of effect might still be of interest. In selecting M_2_, investigators and drug developers should consider consulting with patients to ascertain whether a margin acceptable to regulators is too large to be acceptable to patients.

Expanding the primary endpoint into a composite incorporating efficacy and quality of life, efficacy and cost, or efficacy and safety, would be complicated. We advocate considering whether to revise the relevant legislation to modify the regulations so that regulators are legally able to take into account multiple dimensions of a new product. The resulting analyses would become ever more complicated, but the regulatory decisions would be more nuanced and ultimately better for the public health. At present, however, success in a non-inferiority trial in the USA depends upon success in the primary outcome measure, not on other aspects of benefit, such as safety, and regulatory success using non-inferiority trial designs may require completion of more than one such trial.

## Competing interests

The authors declare that they have no competing interests.

## Authors' contributions

JS and JW drafted the manuscript. Both authors read and approved the final manuscript. We thank the reviewers for helpful comments and suggestions, and Tara Gentile for assistance with creation of the figures.
